# Influence of Reactive Oxygen Species on the Enzyme Stability and Activity in the Presence of Ionic Liquids

**DOI:** 10.1371/journal.pone.0075096

**Published:** 2013-09-16

**Authors:** Pankaj Attri, Eun Ha Choi

**Affiliations:** Plasma Bioscience Research Center, Department of Electrical and Biological Physics, Kwangwoon University, Seoul, Korea; Queen's University Belfast, United Kingdom

## Abstract

In this paper, we have examined the effect of ammonium and imidazolium based ionic liquids (ILs) on the stability and activity of proteolytic enzyme α-chymotrypsin (CT) in the presence of cold atmospheric pressure plasma jet (APPJ). The present work aims to illustrate the state of art implementing the combined action of ILs and APPJ on the enzyme stability and activity. Our circular dichroism (CD), fluorescence and enzyme activity results of CT have revealed that buffer and all studied ILs {triethylammonium hydrogen sulphate (TEAS) from ammonium family and 1-butyl-3-methyl imidazolium chloride ([Bmim][Cl]), 1-methylimidazolium chloride ([Mim][Cl]) from imidazolium family} are notable to act as protective agents against the deleterious action of the APPJ, except triethylammonium dihydrogen phosphate (TEAP) ammonium IL. However, TEAP attenuates strongly the deleterious action of reactive oxygen species (ROS) created by APPJ on native structure of CT. Further, TEAP is able to retain the enzymatic activity after APPJ exposure which is absent in all the other systems.This study provides the first combined effect of APPJ and ILs on biomolecules that may generate many theoretical and experimental opportunities. Through this methodology, we can utilise both enzyme and plasma simultaneously without affecting the enzyme structure and activity on the material surface; which can prove to be applicable in various fields.

## Introduction

The production and applications of enzymes have rapidly increased, not only in biochemical research, while it also affects chemical, food and pharmaceutical industries, due to exquisite biological activities of enzymes [1]–[3]. Enzymes 3D structures have several weak interactions such as ionic effects, hydrogen bonding and hydrophobic interactions which can be disturbed by small changes in the environment of the enzymes [1]–[2]. Such small changes can result in denaturation or inactivation of the enzymes via unfolding [4]–[6]. Denaturation of the enzymes poses a serious problem not only in the separation and storage of enzymes, but also in the process of biotransformation, biosensing, drug production, etc. Several strategies have been proposed so far in order to prevent denaturation of enzymes [7]–[11], which include chemical modification, immobilization, genetic modification and use of stabilizing agents. The addition of stabilizing agents is one of the most convenient and preferred methods for minimizing the denaturation of the enzymes [4]–[11]. Nowadays, ionic liquids (ILs) have emerged as the most popular tool for stabilization of biomolecules [9], [12]–[14]. ILs, have also been recently identified as useful solvents and additives for protein storage, enzymatic reactions and green chemistry [15]–[31]. To our knowledge there has been no previous study regarding the protective action of ILs against reactive oxygen species (ROS) produced by atmospheric pressure plasma jet (APPJ).

APPJ plays an important role in various biomedical applications such as improvement of the material biocompatibility and sterilization of the biological samples [32]–[37]. Moreover, the utilization of atmospheric air not only reduces the complexity and cost of the device but also enhances the production of reactive species such as hydroxyl radicals, atomic oxygen, and nitric oxide [34], [38], [39]. Few experiments examining the APPJ exposure on the DNA molecule for short intervals showed that it leads to DNA damage. APPJ induces the formation of single- and double- stranded breaks in extra-chromosomal plasmid DNA (pBR322, 4361 base pair) extracted from E. coli bacteria. The complexity of plasma-generated species, i.e. excited atoms and molecules, charged particles, electrons and UV light gives a variety of possible pathways by which DNA damage can occur [40], [41]. A similar effect of plasma on the enzyme structure was observed by Dudak *et al.* [42]. Treating enzyme with plasma technology modifies the surface of ovalbumin structure as well as improves its solubility [43]. Additionally, the chemical effects of APPJ on lysozyme in the aqueous solution revealed that plasma treatment decreased enzymatic activity and changed the secondary structure which in turn is due to the increased molecular weight of lysozyme with chemical modification. These effects arise neither from UV light nor from the plasma heat, suggesting that the role of ROS generated by the plasma effect on lysozyme [44]. These experimental results by various research groups [40]–[45] emphasise that the structural modifications in biomolecules on treatment with plasma may vary from biomolecule to biomolecule. However, detailed understanding of the mechanism of action of APPJ on biomolecules in the presence of ILs at the molecular level is missing.

**Figure 1:**
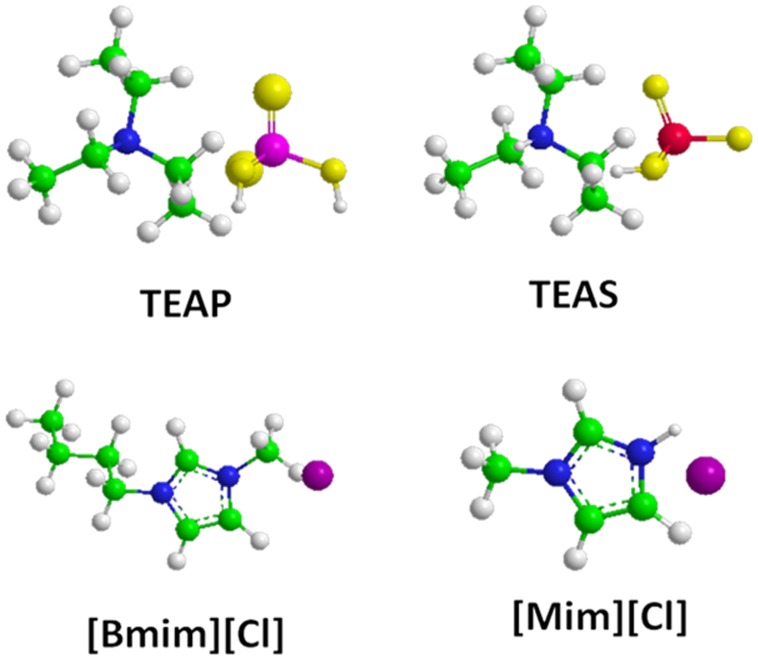
Schematic structures for ILs such as TEAP, TEAS, [Bmim][Cl] and [Mim][Cl].

Recently, combination of the emerging technologies ILs and APPJ is gaining increasing attention. Enders *et al*. [46], [47] reported the combination of cold plasma/IL method for the preparation of nanocrystalline material of Ag. In addition to this, a few research groups [46]–[48] have shown that the combination of ILs and plasma is very promising for various applications in chemical syntheses and nano material fabrication. On the contrary, the combination of ILs and APPJ remains an unexplored area in bioscience. Moreover, we are trying to develop new methodology so that one can use both enzyme and plasma at the same time without affecting the enzyme structure and activity on the material surface. In the light of these considerations, we have specially designed ILs and air-plasma jet device (APPJ) to study the effect on proteolytic enzyme α-chymotrypsin (CT) in the presence of both ILs and APPJ. The mechanism of biocompatible ILs on APPJ–induced denaturation of biomolecules has remained elusive and there is no consensus regarding interpretation of the experimental data. To resolve this dilemma, we have explored the effect of two families of ILs, such as triethylammonium dihydrogen phosphate (TEAP) and triethylammonium hydrogen sulphate (TEAS) from the ammonium family and 1-butyl-3-methyl imidazolium chloride ([Bmim][Cl]), 1-methylimidazolium chloride ([Mim][Cl]) from the imidazolium family on CT in the presence of APPJ. The schematic structure of ILs are as displayed in Figure 1. These results have been analysed using the combination of circular dichroism (CD), fluorescence spectroscopic techniques followed by enzyme activity. This paper presents the first investigation revealing that ILs can protect the enzyme structure and activity against the deleterious action of ROS produced by APPJ.

## Materials and Methods

### Materials

α-Chymotrypsin (CT) from bovine pancreas type II, essentially salt free (molecular weight: 25 kDa) was obtained from Sigma–Aldrich (USA). The imidazolium IL, 1-methylimidazolium chloride ([Mim][Cl], was purchased from Sigma–Aldrich (USA). All materials, with high purity, were used without further purification. Sodium phosphate buffer solution of pH 7.2 was prepared using distilled deionized water at 18.3 Ω.cm. Rest of ILs were synthesized [12], [15], [25] in laboratory, the preparation is given below.

### Synthesis of ILs

#### Synthesis of Triethylammonium Dihydrogen Phosphate (TEAP)

The synthesis of ionic liquids was carried out in a 250 ml round-bottomed flask, which was immersed in a water-bath and fitted with a reflux condenser. Phosphoric acid (1 mol) was dropped into the triethylamine (1 mol) at 70°C for 1 h. The reaction mixture was heated at 80°C with stirring for 2 h to ensure that the reaction had proceeded to completion. The reaction mixture was then dried at 80°C until the weight of the residue was constant. The sample analysed by Karl Fisher titration revealed very low levels of water (below 70 ppm). The obtained yield of TEAP was 98%. ^1^H NMR (DMSO-d_6_): δ (ppm) 1.18 (t, 9H), 3.06 (m, 6H), 6.37 (s, 1H).

#### Synthesis of Triethylammonium Sulphate (TEAS)

The similar procedure as that delineated above for TEAP was followed with the difference involving that sulphuric acid [anion] was used instead of phosphoric acid. The obtained yield of TEAS was 98%. ^1^H NMR (CDCl_3_): δ (ppm) 1.3 (t, 9H), 3.16 (m, 6H), 5.04 (s, 1H).

#### Synthesis of 1-Butyl-3-Methylimidazolium Chloride ([Bmim][Cl])

To a vigorously stirred solution of 1-methylimidazole (1.25 mol) in toluene (125 ml) at 0°C, 1-chlorobutane (1.38 mol) was added. The solution was heated to reflux at 110°C for 24 h, after which it was placed in a freezer at −20°C for 12 h. The toluene was then decanted and the remaining viscous oil/semi-solid was recrystallized from acetonitrile and then again recrystallized from ethyl acetate to yield a white crystalline solid, which was further dried under reduced pressure to give [Bmim][Cl] in approximately 86% yield. ^1^H-NMR (400 MHz, DMSO-*d*_6_): δ (ppm) 10.54 (1H, s), 7.55 (1H, m), 7.40 (1H, m), 4.26 (2H, t, *J = *7.3 Hz), 4.11 (3H, s), 1.82 (2H, m), 1.30 (2H, m), 0.89 (3H, t, *J = *7.3 Hz).

### Measurements

#### Circular dichroism spectroscopy

CD spectroscopic studies were performed using a J-815 spectrophotometer (Jasco, Japan) equipped with a Peltier system for controlling the temperature. CD calibration was performed using (1S)-(+)-10-camphor sulfonic acid (Aldrich, Milwaukee, WI), which exhibits a 34.5 M/cm molar extinction coefficient at 285 nm, and 2.36 M/cm molar ellipticity (θ) at 295 nm. The sample was pre-equilibrated at the desired temperature for 15 min and the scan speed was fixed for adaptative sampling (error F 0.01) with a response time of 1 s and 1 nm bandwidth. The secondary and tertiary structures of CT were monitored by using 1.0 cm path length cuvette. The concentrations for secondary and tertiary structures of CT were 0.1 mg/ml and 1 mg/ml respectively, each spectrum being an average of six spectra. Each sample spectrum was obtained by subtracting appropriate blank media without CT from the experimental enzyme spectrum. The percentages of secondary structures were calculated by using K2D2 online software [49].

Thermal denaturation studies were carried at a heating rate of 1°C/min. This scan rate was found to provide adequate time for equilibration. The sample was heated from 20 to 80°C, where the change in absorbance at 288 nm was observed with increasing the temperature. After denaturation, the sample was immediately cooled down to measure the conformational changes of the protein. The error in melting temperature (T_m_) did not exceed 0.1°C. The estimated relative uncertainties in (ΔH), (ΔC_p_) and (ΔG_U_) were around 2–5% of the reported values [50].

#### Fluorescence spectroscopy

Steady-state fluorescence measurements were conducted with a Cary Eclipse spectrofluorimeter (Varian optical spectroscopy instruments, Mulgrave, Victoria, Australia) equipped with thermostat cell holders and temperature was kept constant by a circulating water bath using a Peltier device attached to the sample holder of the fluorimeter [9]. The excitation wavelength was set at 290 nm to evaluate the contribution of the tryptophan residues to the overall fluorescence emission. The experiments were performed at 25°C by using a 1 cm sealed cell and both excitation and emission slit width were set at 5 nm, and corrected for background signal. Both the change in fluorescence intensity and the shift in fluorescence maximum wavelength were recorded to monitor the unfolding transition; each spectrum being an average of six spectra.

#### Enzyme activity

The activity of CT was measured by the method of Erlanger et al., using SAPNA (Suc-Ala-Ala-Pro-Phe-p-nitroanilide) as a substrate [51]. 0.1 mM SAPNA (Sigma-Aldrich Co. St. Louis, MO) was dissolved in 50 mM Tris-HCl pH 8.2, and 20 mM CaCl_2_ as substrate solution. A substrate solution about 100 ml was introduced in a beaker which contained 63 mg SAPNA and 10 ml of buffer and this mixture was gently stirred for 3 h at the room temperature. By this method, we prepared the stock solution for obtaining the CT activity in 1 M ILs. In order to obtain enzyme activity, we added 1 ml of stock solution to 9 ml of the buffer and mixed thoroughly. Activity was measured by a UV-VIS spectrophotometer (Shimadzu, UV- 1601, Tokyo, Japan), kinetic function using the spectrophotometer was selected and the absorbance was set at 410 nm and kinetic time as 3 min, and the course of reaction was monitored after each 30 sec. 10 µl of sample was transferred to 1 ml quartz cuvette and 750 µl of substrate was added to read the absorbance at 410 nm/min. Specific activity of CT per ml of sample was calculated using the following equation.
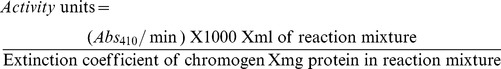
(1)


The molar extinction coefficient of para-nitroanilidine liberated from chromogens of SAPNA is 8800 M^−1^ cm^−1^.

#### Atmospheric Pressure Plasma Device (APPJ)

The key components of cold atmospheric plasma jet system were electrodes, dielectrics, and a high-voltage power supply. The ac power supply was a commercially available transformer for neon light operated at 60 Hz and was connected to two electrodes. The voltage controller regulated the primary voltage of the high voltage transformer. The inner electrode was a typical injection needle made of stainless steel with an inner diameter of 1.2 mm and a thickness of 0.2 mm; it was tightly covered with a quartz tube with an outer diameter of 3.2 mm. Porous alumina 10 mm in diameter and 20 mm in length was machined for the inner electrode, through which the quartz tube was inserted. The tip of the inner electrode and the inner surface of the porous alumina were placed in contact. The outer electrode was fabricated from stainless steel and had a somewhat conical shape; it was centrally perforated with a hole of 1 mm through which the plasma jet was ejected to the surrounding ambient air. The porous alumina with the inner electrode was installed within the outer electrode. The discharge gap (*dg*) was the distance (in this work, *dg* is 2 mm) between the tips of the porous alumina and the inner electrode. It could be adjusted by controlling the depth at which the inner electrode was inserted into the porous alumina. The inner surface of the outer electrode and the tip of the porous alumina were also in contact. Air was injected into the injection needle through 1 mm hole in the outer electrode *via* the porous alumina. The alumina used in this work had approximately 30% vol. porosity and an average pore diameter of 100 µm. Once air was introduced through the inner electrode and high-voltage ac. power was applied, a discharge was fired in the porous alumina between the electrodes and a long plasma jet reaching lengths up to several centimetres was ejected to the open air. We have used the compressed atmospheric dry air with purity of 99.9999%. This dry air had a flow rate of 3 liter/min, which could be adjusted and measured by mass flow controller.

**Figure 2:**
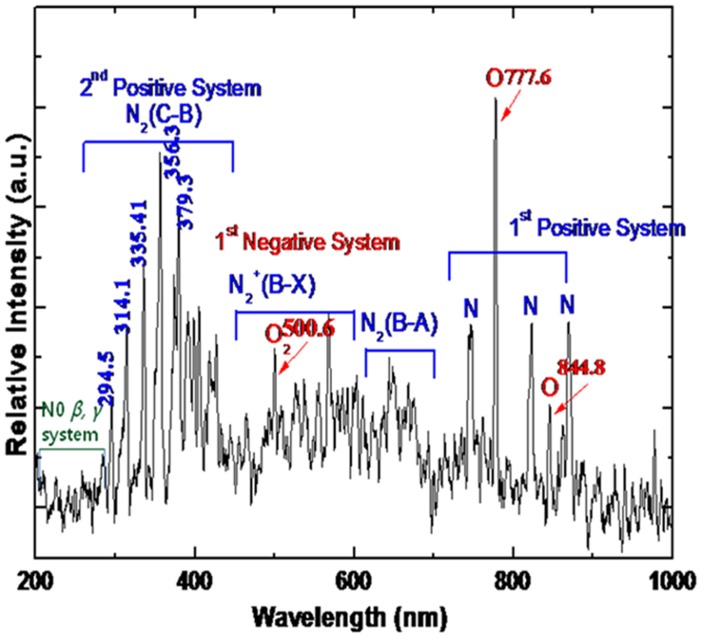
The emission spectra of cold atmospheric-pressure plasma jet (APPJ).

The plasma parameters of electron temperature and plasma density have been measured. In the measurement of electron temperature, a high speed image intensified camera was used to get the temporal propagation distance versus elapsed time in our experiment. Once we had average value of temporal distance Δx versus time interval Δt, the average plasma propagation speed for ionization front could be obtained by 
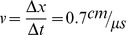
. This plasma speed could be expressed by 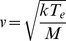. Here the electron temperature kT_e_ could be experimentally measured by ∼1.7 eV under dry air gas. Also the plasma density could be obtained from 
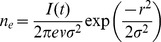
 where I is measured electric current, e is electron charge, 
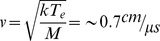
 is plasma propagation velocity determined by high speed image camera, and σ is radial profile of plasma jet determined by experiment. From these parameters, the electron temperature 1.7 eV and plasma density 3.3×10^12^ cm^−3^ could be estimated respectively. The ionization rate is about 10^−7^, so that this plasma belongs to cold plasma which has almost room temperature since most of air molecules are not ionized but in neutral state. The emission spectra was recorded as illustrated in Figure 2. We have observed the emission lines from molecular NO *β*, γ system between 200 and 250 nm and superoxide anion O_2_* of 245 nm as well as the emission line of 307 nm from OH molecules.

It is noted in this experiment that the VUV lines in the range 120 nm - 200 nm are easily absorbed by the atmospheric oxygen molecules; however, the emission lines beyond 200 nm are easily passed through the oxygen molecules in the atmospheric air, which enables us to investigate the emission spectrum between the 200 nm and 1000 nm. We observed that the emission spectra emitted from the molecular N_2_ (C-B) second positive system could be seen at the wavelengths between 300 nm and 400 nm. Also lines from molecular N_2_^+^ (B-X) are also shown between 450 nm and 600 nm, and those for N_2_ (B-A) are shown between 600 nm and 700 nm. It is also seen that the emission lines from the O_2_ first negative system are observed at the wavelengths between 470 nm and 620 nm, where typical 500.6 nm is observed in this experiment. The spectra of N_2_ molecular first positive system appeared from 700 nm and 900 nm. Also, it is necessary to mention that the oxygen atom O lines of 777. 6 nm and 844.8 nm could be observed in this experiment.

#### Sample preparation

Enzyme stability was analyzed by incubating 2 ml screw-capped vials in 10 mM sodium phosphate buffer pH 7.2 solutions in the presence or the absence of co-solvents (TEAP, TEAS, [Bmim][Cl] and [Mim][Cl]) at 25°C for 4 h to attain complete equilibrium. All samples were prepared at 1 mg/ml enzyme concentration in 1 M concentrations of co-solvent for thermal CD experiments. After completely dissolving the enzyme in the solution, the mixture was filtered with a 0.45 µm disposal filter (Millipore, Millex-GS) through syringe before performing the measurements. After incubation of CT with or without co-solvent for 4 h, the samples were treated at 23 mm distances from the APPJ tip for 5 min. Three samples were treated for each condition to minimize the error.

## Results and Discussion

The stability of proteins in ILs is a most interesting and important emerging scientific research area while the APPJ is beginning to play its role in bioscience. To obtain more detailed and new delineation of the combined role of ILs and APPJ on the stability of enzyme, we have performed the CD spectroscopy, fluorescence spectroscopy experiments followed by determining enzyme activity.

### Thermal Analysis Using CD Spectroscopy

The thermodynamic profile at wavelength 288 nm based on the ellipticity, which is caused by alteration in the local environment of aromatic residues and such studies will help in exposing the melting of the CT occurring in a single step reaction (with simultaneous loss of both secondary and tertiary structure) [8], [52], [53]. Thermodynamic parameters can be related more directly to the structural arrangements of enzymes and to their interactional solvent effects, which help in anticipating the contributions of protein stability. The elementary thermodynamic forces that underlie the structure and function of biomolecules are the quintessence for chemical technology. Explicitly, Gibbs free energy change (Δ*G_U_*) is useful to describe the global protein folding studies, while the enthalpy change (Δ*H*), heat capacity change (Δ*C*_p_), and melting temperature (*T*_m_) are useful in evidencing the protein stability in terms of non-covalent forces of the different structural states.

**Figure 3:**
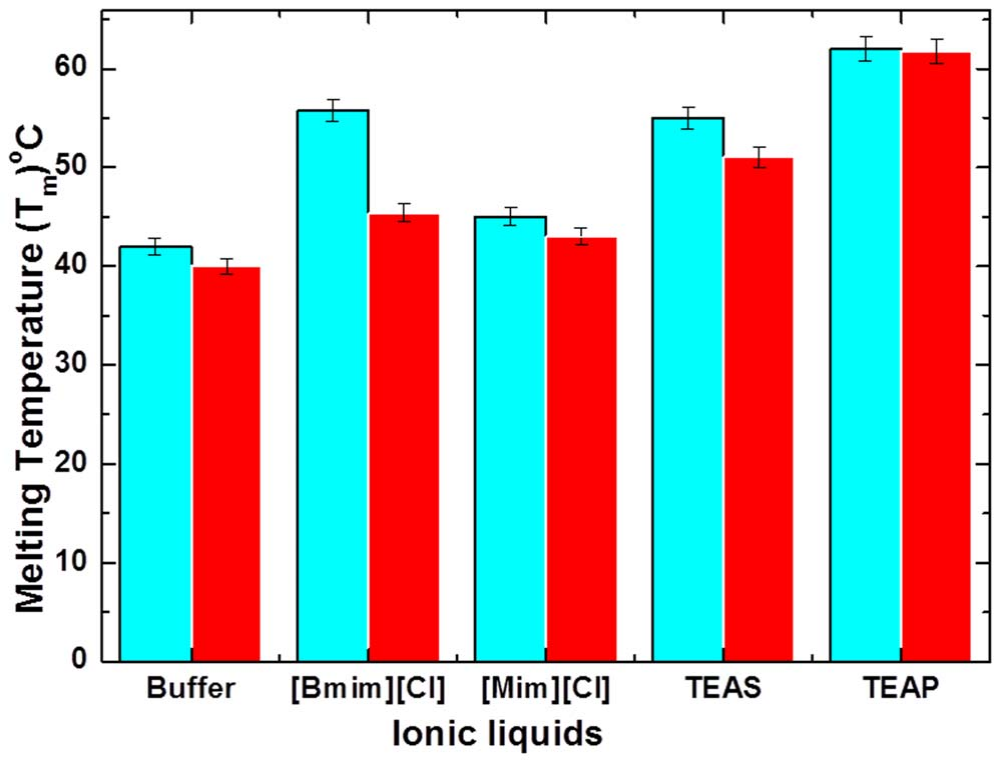
The variation in melting temperature (T_m_) values of CT in buffer and ILs (a) without APPJ treatment (cyan colour) and (b) with APPJ treatment (red colour). The data points are average values of atleast three determinations, the error bars indicating ± mean deviation.

In addition to this, all ILs used in our study stabilize the native structure of CT without APPJ action. In the presence of co-solvents, we have used ILs to investigate the effect of APPJ on CT. We have used 1 M TEAP, 1 M TEAS, 1 M [Bmim][Cl] and 1 M [Mim][Cl] as ILs. After the treatment of CT with APPJ for 5 min, there is a variation in the thermodynamic profile of CT in the presence of ILs, as illustrated in Table 1. The ΔG_U_ value explains the stability of the enzyme in the folded state, hence after APPJ exposure, there is decrease in ΔG_U_ value for CT in ILs. The ΔG_U_ value results show that the stabilizing action of ILs against APPJ exposure varies from IL to IL, thereby the efficiency of stabilization follows the trend TEAP> TEAS>[Bmim][Cl]>[Mim][Cl] as illustrated in Table 1. Interestingly, T_m_ of the folding induced by the ILs reveals that TEAP is the strongest stabilizer, TEAS or [Bmim][Cl] is a moderate stabilizer and [Mim][Cl] is a weak stabilizer, as shown in Figure 3. Therefore, our studies clearly affirm that all ILs have thermodynamic profile of CT structure higher than buffer after APPJ exposure. While amongst all ILs, TEAP is able to counteract strongly against the APPJ action, due to highly unfavourable interaction of TEAP with the functional groups of CT.

**Table 1:** Melting temperature (T_m_), enthalpy change (ΔH), and heat capacity change (ΔC_p_) are determined by CD and calculated Gibbs free energy change (ΔG_U_) in unfolding state at 25°C for the CT in different media^a^.

Sample	T_m_ (°C)	ΔH (kJ.mol^−1^)	ΔG_U_ (kJ.mol^−1^)	Δ C_p_ (kJ.mol^−1^.C^−1^)
Buffer	42.0	424	18.2	10.0
Buffer+AAPJ	40.0	299	11.7	7.1
1 M TEAP	62.0	842	57.4	14.0
1 M TEAP+AAPJ	61.8	840	57.2	13.6
1 M TEAS	57.0	688	44.3	12.1
1 M TEAS+AAPJ	51.1	613	33.7	12.0
1 M [Bmim][Cl]	55.8	652	43.6	11.7
1 M [Bmim][Cl]+AAPJ	45.4	546	26.9	12.0
1 M [Mim][Cl]	45.0	428	20.8	9.5
1 M [Mim][Cl]+AAPJ	43.0	394	17.6	9.2

a The estimated relative uncertainties in (ΔH), (ΔC_p_) and (ΔG_U_) are around 2% of the reported values.

### CD Analysis for the Secondary and Tertiary Structure

**Figure 4:**
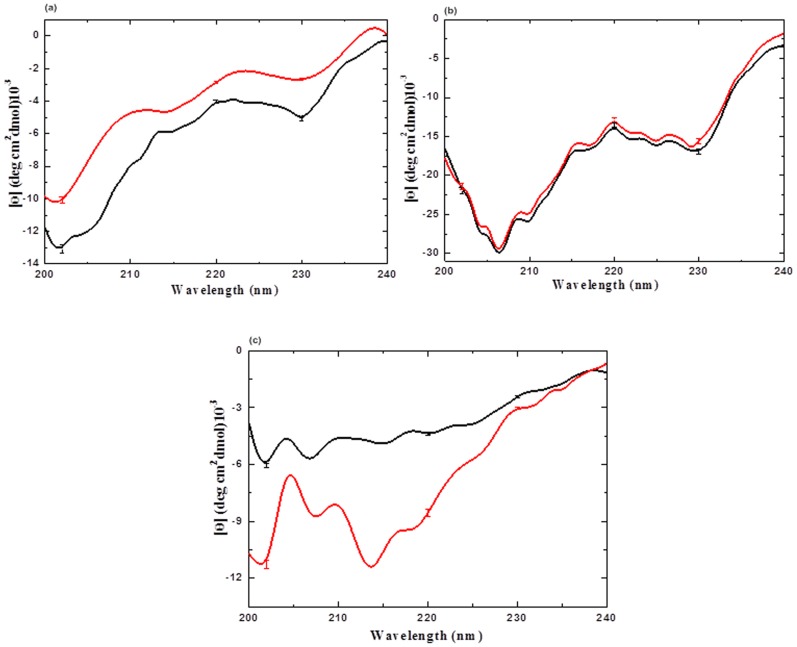
Far-UV CD spectra analysis of CT in (a) buffer, (b) 1 M TEAP and (c) 1 M TEAS at 25°C, where the ILs are used without treatment with AAPJ for 5 min are in black colour and the treated ILs are in red colour. The data points are average values of atleast six determinations, the error bars indicating ±mean deviation.

To study the role of ILs (before and after the treating with APPJ) in enhancing the stability of CT structure we further proceeded with CD spectrum analysis. The far-UV CD spectrum (200–240 nm) of the enzyme indicated that CT has a peculiar secondary structure in presence of all of the above co-solvents discussed. CT is a type of all-β protein characterized by a CD spectrum, which resembles that of a random coil conformation [29], [54]. Crystal structure data showed that this kind of protein consists of antiparallel pleated β-sheets which are either highly distorted or form very short irregular strands [29].The same was reflected with APPJ action as well. The CD spectrum of CT in buffer, TEAP and TEAS displayed no positive bands in entire range (200–240 nm), as illustrated in Figure 4. While in case of imidazolium ILs ([Bmim][Cl] and [Mim][Cl]), we observed a positive CD peak with high ellipticity (data not shown here). The obtained far-UV CD spectrum (200–240 nm) of the CT in buffer is very well sustained with earlier studies [29], [49]. CD results of CT in the presence of TEAP and TEAS are very well correlated with earlier studies (by T. de Diego, *et al.)* investigating the stability of CT in presence of IL [29]. This shows that the denaturation action significantly increases on the plasma treatment in buffer, which is attributed to the loss of secondary structure of CT (Figure 4a). The far-UV CD spectra data help us to estimate β-structure using K2D2 software, and these results are shown in Table 2. The secondary structure contents of CT in buffer are very well correlated with previous studies (8% α-helix, 35% β-sheet and 57% random coil) [29], [49]. Table 2 shows that in the presence of TEAS, there is slight loss of secondary structure of CT after treatment of APPJ; this might be due to hydrogen-bonding rearrangements occurring due to APPJ action. However, TEAP is capable of hindering the denaturation action due to APPJ effect, which is responsible for sustainance of the secondary structure conformation of CT in native state even on treatment with APPJ. On comparing the CD signals before and after treatment with APPJ, we found that among TEAP and TEAS, only TEAP followed the same ellipticity trends, illustrated in Figure 4 and Table 2. From Table 2, we can observe that there is negligible change in α, β and random coil % of structure in the presence of TEAP before and after treatment.

**Table 2:** Secondary structure composition of CT determined from Far UV CD spectra in different media at 25°C determined by K2D2.

Sample	α-Sheet (%)	β-Sheet (%)	Random (%)
Buffer	8	35	57
Buffer+APPJ	10	33	57
1 M TEAP	2	51	47
1 M TEAP+APPJ	3	48	49
1 M TEAS	6	48	46
1 M TEAS+APPJ	8	42	50

**Figure 5:**
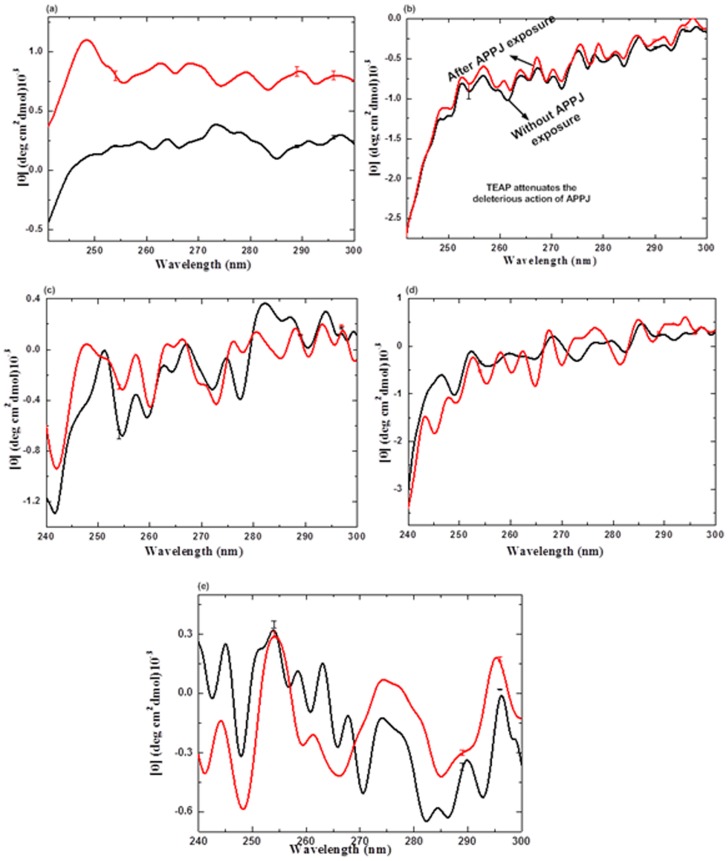
Near-UV CD spectra analysis of CT in (a) buffer, (b) 1 M TEAP, (c) 1 M TEAS, (d) 1 M [Bmim][Cl] and (e) 1 M [Mim][Cl] at 25°C, where the ILs are used without treatment with APPJ for 5 min are in black colour and the treated ILs are in red colour. The data points are average values of at least six determinations, the error bars indicating ± mean deviation.

Further, we ascertained the CD spectrum in near-UV region (240–300 nm), for investigating the variations in tertiary structure of CT due to the effect of APPJ in absence as well as presence of ILs; the results are illustrated in Figure 5. The dominant contribution of aromatic residues of proteins (Tyr and Trp) to the CD spectrum is well exhibited in near-UV region and disulfide bond which can act as a chromophore contributes to the usually weak and broad signals. The local conformational changes around the chromatophores are responsible for the variations in the intensity of the 240–300 nm bands. The near-UV CD spectrum of CT in buffer in absence and presence of ILs clearly reflects the significant contribution of Tyr and Trp residues, which is well characterized by the peaks and shoulders at wavelength in between 270–300 nm, and strong absorption bands in the 258–270 nm regions attributed due to the Phe residues. Figure 5a shows the CD spectrum of CT in buffer solutions depicting distinguished maxima at 254, 289 and 296 nm, corresponding to the presence of Trp residues of CT. These results are very well correlated with earlier reported results by various research groups [29], [49], [54]. Decrease in intensity of peaks can be probably due to the treatment of CT with plasma jet; changes being more prominent at 286 nm. Figure 5 shows that no other IL, except TEAP is able to offset the APPJ action on CT. Hence, we can conclude that no change is observed in the structural conformation of CT in TEAP on treatment with APPJ from CD results. Further to confirm these results, we have performed the fluorescence studies.

### Fluorescence Analysis

**Figure 6:**
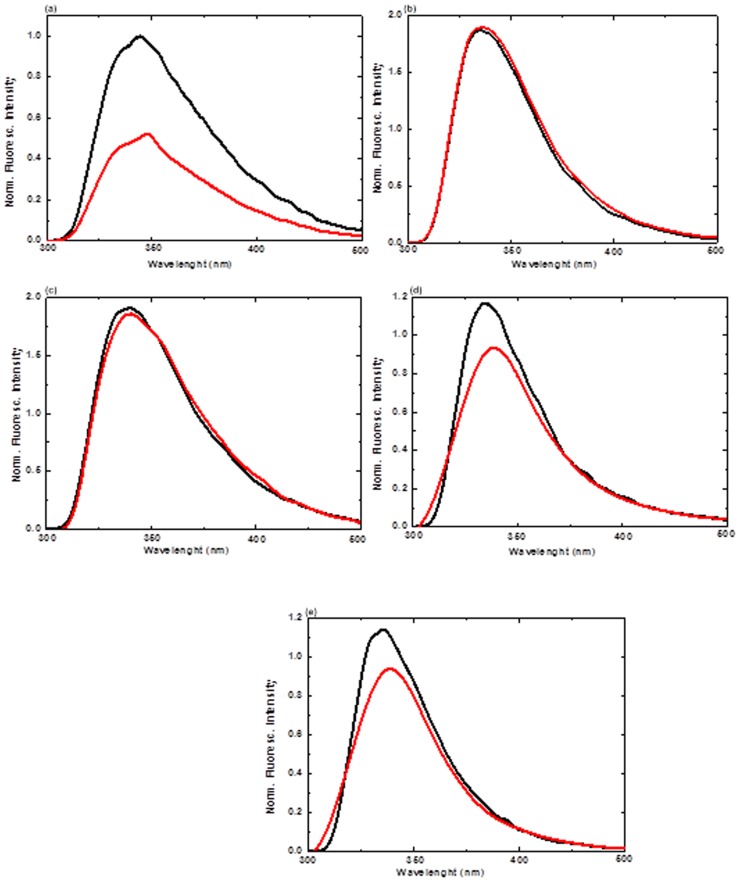
Fluorescence spectra analysis of the CT in (a) buffer, (b) 1 M TEAP, (c) 1 M TEAS, (d) 1 M [Bmim][Cl] and (e) 1 M [Mim][Cl] at 25°C, where the ILs are used without treatment with AAPJ for 5 min are in black colour and the treated ILs are in red colour. The data points are average values of at least six determinations, the error bars indicating ±mean deviation.

To further explore this ability of ILs to counteract the denaturing effect of APPJ on CT, we used the fluorescence spectroscopy to probe the environment of the fluorophore residues. So, we proceeded with the fluorescence spectroscopy to investigate the environment of the fluorophore residues (e.g., Trp, Tyr, or Phe) of the enzyme as illustrated in Figure 6. The denaturation process can be studied by observing changes in the maximal intensity of fluorescence (I_max_) and in the maximal emission wavelength (E_max_) as well as the increased polarity of the Trp residues of the protein [29], [49]. Trp residues have a strong Stokes shift, depending upon the solvent environment. As the co-solvents change, there is change in the maximum emission wavelength of Trp which indicates that E_max_ depends on the co-solvent environment. Table 3 shows the initial fluorescence spectra of native CT on APPJ treatment of CT after 5 min in all the assayed media (buffer, 1 M TEAP, 1 M TEAS, 1 M [Bmim][Cl] and 1 M[Mim][Cl]) at 25°C. Maximum intensity for all the spectra was normalized with respect to the spectrum obtained for the native CT in buffer at 25°C. As can be seen, the enzyme exhibited same initial E_max_ ≈ 344.5 nm in buffer (Table 3), whereas in the presence of other co-solvents such as 1 M TEAP, 1 M TEAS, 1 M [Bmim][Cl] and 1 M[Mim][Cl], it was found to be ≈ 341.0 (Figure 6). After treatment with APPJ for 5 min, it was noticed that the fluorescence spectra of the CT showed changes for both E_max_ and I_max_ parameters as compared to untreated CT in all co-solvents. After APPJ treatment of CT, red shifts were evidenced in all media except TEAP (as illustrated in Table 3) whereas in case of TEAP, we observed no changes in E_max_ and I_max_ parameters. This suggests that after the APPJ treatment of CT in presence of TEAP, there is more stabilization in the structure of CT. In this way, the evolution of both I_max_ and E_max_ in aqueous media corresponds to an usual unfolding process of CT, which enhances the exposure of Trp residues to the bulk solvent (e.g., the E_max_ of free Trp in aqueous solution is 350 nm) [29]. In the hydrophobic environment (buried within the core of the protein), Trp has a high quantum yield and therefore a high fluorescence intensity was observed. In contrast, in hydrophilic environment (exposed to solvent), their quantum yields decreased owing to which low fluorescence intensity was observed. Hence, fluorescence analysis also supports the CD spectral data that evidenced a change in native CT conformation of the treated samples in the presence of ILs (except TEAP). In addition, enzyme activity is seems an important tool to explore the activity of enzyme before and after plasma treatment.

**Table 3:** Fluorescence intensity of the CT in co-solvents with or without APPJ exposure for 5 min.

S. No	Co-solvents	FluorescenceIntensity (au)
1	Buffer	344.5
2	Buffer+APPJ	346.0
3	1 M TEAP	340.0
4	1 M TEAP+APPJ	341.0
5	1 M TEAS	341.0
6	1 M TEAS +APPJ	344.0
7	1 M [Bmim][Cl]	342.0
8	1 M [Bmim][Cl]+APPJ	347.0
9	1 M [Mim][Cl]	341.2
10	1 M[Mim][Cl]+ APPJ	345.6

### Enzyme Activity

To ascertain whether the ILs cause any alternation in the enzyme activity after the plasma treatment of the folding transition state of CT, we further performed enzyme activity experiments [8], [29], [49]. Very few studies are available in the literature [29] related to CT activity in ILs. Therefore, we tested the CT activity in the presence of ILs with and without plasma by using an UV-Vis spectrophotometer. The obtained results are collected in Table 4. It is quite clear from Table 4 that CT in buffer losses its enzyme activity to higher extent after plasma treatment of CT. Our results are very well supported by *Takai et al.*, where they observed the loss of activity of lysozyme after APPJ treatment [44]. Our previous experimental results (CD and fluorescence) show that, among all the ILs, TEAP is a powerful stabilizer and the most compatible of IL, therefore even after the plasma treatment its activity is higher than other ILs (Table 4). This phenomenon explains that CT activity is not significantly decreasing in TEAP as compared to other ILs. Our results are consistent with the experimental results that TEAP can sustain the activity of CT even after the plasma exposure.

**Table 4:** Activity of CT by using 0.1-ala-ala-pro-phe-p-nitroanilide (SAPNA) in various media in the presence and absence of APPJ exposure for 5 min.

Sample	A410 nm/min	CT Concentration(mg/ml)	CT in ReactionMixture (mg/10 µl)	Specific Activity(unit/mg)	Relative SpecificActivity of CT (%)
Buffer	0.6647	0.2553	0.0025	22.4839±0.0081	100.00
Buffer+APPJ	0.0092	0.3064	0.0031	0.2593±0.0026	1
TEAP	1.1085	0.6765	0.0067	14.1493±0.0078	62
TEAP+APPJ	0.965	0.6340	0.0063	13.1443±0.0072	58
TEAS	0.7102	0.5915	0.0059	10.3696±0.0063	46
TEAS+APPJ	0.5679	0.5531	0.0055	8.8659±0.0013	39
[Bmim][Cl]	0.3245	0.5617	0.0056	4.9893±0.0092	22
[Bmim][Cl]+APPJ	0.1759	0.5574	0.0056	2.7252±0.0083	12
[Mim][Cl]	0.5120	1.0425	0.0104	4.2413±0.0058	18
[Mim][Cl]+APPJ	0.0392	0.7659	0.0076	0.4419±0.0074	2

**Figure 7:**
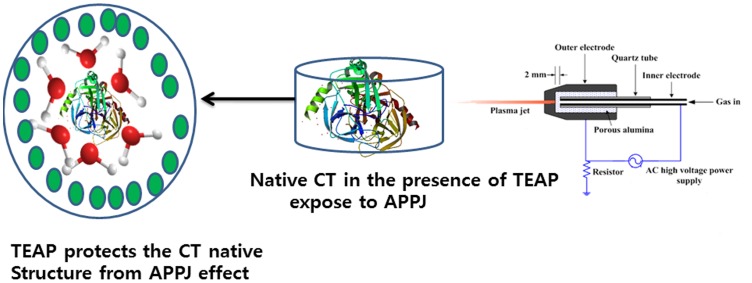
Schematic depiction of TEAP action on CT structure against APPJ exposure.

Our experimental results from CD, fluorescence and enzyme activity suggest that ILs acts as preferentially excluded co-solvents that tend to stabilize the native state of CT [29]. The magnitude of preferential interaction of CT in ILs is correlated with the influence of ILs on physicochemical properties of solution. These physicochemical properties are responsible for the protective action of ILs against the ROS radicals created by APPJ, which may be due to the fact that all ILs (except TEAP) are not able to stabilize the compact structure of CT after treatment with APPJ. Whereas, there is no significant structural changes were observed for CT in TEAP even after APPJ exposure. In other words, TEAP results in stronger interaction with the protective water shield around the CT. As a result, ROS radicals or other charged species are not able to penetrate the shielding created by TEAP around CT. This phenomenon could be basis of a probable mechanism for TEAP IL being able to protect CT activity and structural entity even after APPJ treatment. The same as illustrated in Figure 7.

## Conclusion

The present work confirms that the combination of plasma and ILs provides new opportunities in bioscience research. Our experimental results illustrate that ammonium IL TEAP is a strong stabilizer compared to imidazolium ILs for CT with or without APPJ action. It is affirmed that TEAP is strongly counteracting the detrimental effects of ROS created by APPJ on CT. Furthermore, employing TEAP, the enzyme is able to retain its activity even after APPJ exposure, while the rest of the systems fail to do so. Through this methodology, we can utilise both enzyme and plasma simultaneously without affecting the enzyme structure and activity. It is hoped that the results obtained from the above study will be useful in recommending tailor-made ILs for various applications in biological systems, along with the combination of cold atmospheric plasma that provide challenges to physicists, chemists and biologists.
